# Meat Quality, Amino Acid, and Fatty Acid Composition of Liangshan Pigs at Different Weights

**DOI:** 10.3390/ani10050822

**Published:** 2020-05-09

**Authors:** Mailin Gan, Linyuan Shen, Lei Chen, Dongmei Jiang, Yanzhi Jiang, Qiang Li, Ying Chen, Guihua Ge, Yihui Liu, Xu Xu, Xuewei Li, Shunhua Zhang, Li Zhu

**Affiliations:** 1 College of Animal Science and Technology, Sichuan Agricultural University, Chengdu 611130, China; ganmailin@stu.sicau.edu.cn (M.G.); shenlinyuan@sicau.edu.cn (L.S.); chenlei815918@sicau.edu.cn (L.C.); jiangdm@sicau.edu.cn (D.J.); xuewei.li@sicau.edu.cn (X.L.); 2 Farm Animal Genetic Resources Exploration and Innovation Key Laboratory of Sichuan Province, Sichuan Agricultural University, Chengdu 611130, China; 3 College of Life Science, Sichuan Agricultural University, Yaan 625014, China; 13526@sicau.edu.cn; 4 Sichuan Province General Station of Animal Husbandry, Chengdu 611130, China; B20050604@stu.sicau.edu.cn (Q.L.); S20163634@stu.sicau.edu.cn (Y.C.); S20163617@stu.sicau.edu.cn (G.G.); B20141408@stu.sicau.edu.cn (Y.L.); S20163644@stu.sicau.edu.cn (X.X.)

**Keywords:** Liangshan pig, meat quality, amino acid, fatty acid, traditional pig products

## Abstract

**Simple Summary:**

The research on the quality of traditional pork can not only provide a reference for the thorough breeding and food development of pigs, but also make a reference for understanding the local history and social culture. The Liangshan pig is a traditional Chinese miniature pig breed. It is mainly raised in the Liangshan Yi area and is closely related to the dietary culture of the local people. The characteristics of, and changes in, the meat quality, amino acid composition and fatty acid composition of Liangshan pigs of different weights were revealed for the first time in this paper. It was found that as the weight of Liangshan pigs increased, the contents of marbling score, intramuscular fat, shear force, Met, Asp, Asn, C18: 0 and C20: 2 increased, and drip loss, Trp and C22: 6 decreased. Taken together, our findings serve as a reference for the development of the local Liangshan pig industry.

**Abstract:**

Indigenous pig breeds are important biological resources and their diversity has been severely damaged. The Liangshan pig is a typical mountain-type local pig breed in southwest China. Here, the meat quality, amino acid, and fatty acid composition of Liangshan pigs were compared at seven stages within the weight range of 50–90 kg. A score for comprehensive factors of meat quality was maintained after rising and kept in a plateau within 74.9–91.5 kg of body weight. The total amount of amino acids in the longissimus dorsi muscle remained stable, and the total fatty acids showed an upward trend. Amino acid composition analysis revealed that as the body weight of Liangshan pigs increased, umami, basic, and acidic amino acid contents decreased, while the essential amino acids (EAA) content and the ratio of basic amino acids to acidic amino acids increased. Fatty acid composition analysis revealed that as body weight increased, the content of polyunsaturated fatty acids (PUFA) exhibited a downward trend, while the content of saturated fatty acids (SFA) exhibited an upward trend. This study is a primary step towards the development and utilization of Liangshan pigs and provides useful information for local pork processing and genetic improvement.

## 1. Introduction

Local pigs are important biological resources for new breeds and strains, the protection of animal diversity, and the realization of sustainable animal husbandry [1]. Pig production and breeding have rapidly entered globalization alongside economic globalization. Duroc, Yorkshire, Landrace, and Buckshire pigs represent the majority of breeds on the market, whereas many local pig breeds are endangered [2]. It is worth noting that pig breeding has long pursued high growth rates and high lean meat rates, which has led to a decline in pork quality, such as meat color, shear force and flavor [3]. However, consumers have recently begun to pursue pork of a higher quality and richer flavor. Therefore, local pig breeds are a resource that could meet the diverse needs of consumers [4].

The formation of local pig breeds is closely related to the local environment and people’s consuming habits [5]. Pigs are an important part of local society and culture. In-depth studies of pork quality not only provide a reference for improved breeding and food development but can also provide insights into local history and social culture. The development and utilization of local pig breeds is an important way to protect local pig resources and diet culture and is of great significance for local economic development and national cultural heritage.

The Liangshan pig is a traditional small-sized Chinese indigenous pig breed, mainly reared in the Yi minority region of Liangshan, China. It has a strong resistance to cold and thrives on coarse feed. Like most local pigs, Liangshan pigs have strong adaptability and good meat quality. However, Liangshan pigs have a slow growth rate and low feed conversion rate; therefore, the population of Liangshan pigs has decreased rapidly in recent years [6]. Limited information exists on the Liangshan pig breed; therefore, the goal was to acquire basic information of different quality characteristics to be used a future reference in the development and utilization.

In the present study, the quality, and amino acid and fatty acid composition of meat from 140 slaughtered Liangshan pigs was measured. The analysis of these data will help towards understanding meat quality characteristics and change rules of Liangshan pigs, and to formulate optimal slaughter times and suitable food development strategies. The results of this study are also of reference value for the genetic improvement of other local pigs and the development of specialty foods.

## 2. Materials and Methods

The experimental protocol was approved by the Animal Care and Ethics Committee of Sichuan Agricultural University, Sichuan, China, under permit No. DKY-S20123030 and No. DKY-S20123138.

### 2.1. Animals

The experiment was organized and performed at the Liangshan pig conservation farm of Mabian Gold Liangshan Agriculture Development Co. Ltd. (Sichuan, China). A total of 140 pigs with a similar birth date and birth weight (half barrows and half gilts) were randomly selected from the farm. Based on the methods of previous reports, 140 pigs were slaughtered at 7 different weight stages (the difference between each stage was approximately 6 kg) between 160 and 260 days of age, with 20 pigs from each stage (Table 1). The ingredients of the basal experiment diets are shown in Table S1.

### 2.2. Management

All pigs were fed the same commercial feedstuff. The pigs had ad libitum access to diet and water. All pigs were slaughtered following the method of Xiao et al. [7]. After transport to the abattoir, the pigs had no access to feed for 24 h before slaughter.

### 2.3. Meat Quality Trait Measurements

The determination of meat quality traits mainly refers to those used in our previous study [1]. The longissimus dorsi muscle samples used to measure meat quality traits were collected from the left side of the carcass adjacent to the last rib, within 45 min after slaughter. The penultimate 3–4 intercostal samples (the thickness is about 3 cm) of the longissimus dorsi muscle were used to measure pH, color and marbling scores (MS), and the samples (about 300–500 g) of the last rib of the longissimus dorsi muscle was used to measure drip loss, cooking loss and shear force (SF). The meat samples’ pH was determined using a pH meter (model 720A; Orion Research Inc., Boston, MA, USA) according to the procedure of Alonso et al. [8]. The first measurement was to measure the central 1/3 location area of the meat sample at 45 min post-mortem (pH_1_), and the second at 24 h (pH_2_). Make 3 repetitions for each sample, take 3 readings for each repetition, and then calculate the average. Color parameters were measured using a Minolta CR-300 colorimeter (Minolta Camera, Osaka, Japan). Drip loss was calculated from the weight loss of a sample (approximately 30 g) wrapped in foil and placed on a flat plastic grid after storage for 24 h at 4 °C. Cooking loss was determined by cooking meat samples for 30 min, then, after cooling, measuring the weight loss relative to the uncooked weight. Marbling scores (MS) were determined using longissimus dorsi muscle 24 h after slaughter (colorimetric method, 5-point scale; the larger the score value, the richer the muscle fat content). Shear force (SF) was determined using a Texture Analyzer (TA.XT. Plus, Stable Micro Systems, Godalming, UK) equipped with a Warner-Bratzler shearing device.

### 2.4. Analysis of Free Amino Acids and Fatty Acids

Free amino acid (FAA) and fatty acid compositions were determined according to a previous article [1]. FAA composition was measured using liquid chromatography–mass spectrometry (Liquid phase: LC-20AD, Shimadzu, Japan; Mass Spectrometry: 5500 Q TRAP LC-MS/MS, AB SCIEX, Framingham, MA, USA), and gas chromatography–mass spectrometry (GC-MS 7890B-5977A, Agilent, Palo Alto, CA, USA) was used to detect fatty acid composition.

### 2.5. Meat Chemical Composition

Intramuscular fat (IMF), crude protein (CP) and ash contents were measured by the Nutrition Institute of Sichuan Agricultural University. CP was determined by the Kjeldahl method, and IMF content was determined by Soxhlet extraction [9].

### 2.6. Statistical Analyses

The ANOVA procedure was performed in SAS for Windows Release 8.0 (SAS Institute Inc., Cary, NC, USA) and was used to analyze the data collected. Duncan’s test was used for comparing the mean values of the results. Mean values and standard errors are shown in the tables, with differences considered significant if *p* < 0.05. A comprehensive evaluation of Liangshan pigs at different bodyweight stages was performed using a factor analysis test.

## 3. Results

### 3.1. Meat Quality and Meat Crude Chemical Composition

The meat quality and crude chemical composition of Liangshan pig meat samples exhibited significant differences at different stages (Table 2). The first stage exhibited the highest L*_2_ and drip loss values, while the marbling score, shear force, crude protein, and intramuscular fat content were the lowest. At the seventh stage, meat samples’ pH_1_, shear force, and intramuscular fat content were the highest, while drip loss and ash value were the lowest.

**Figure 1:**
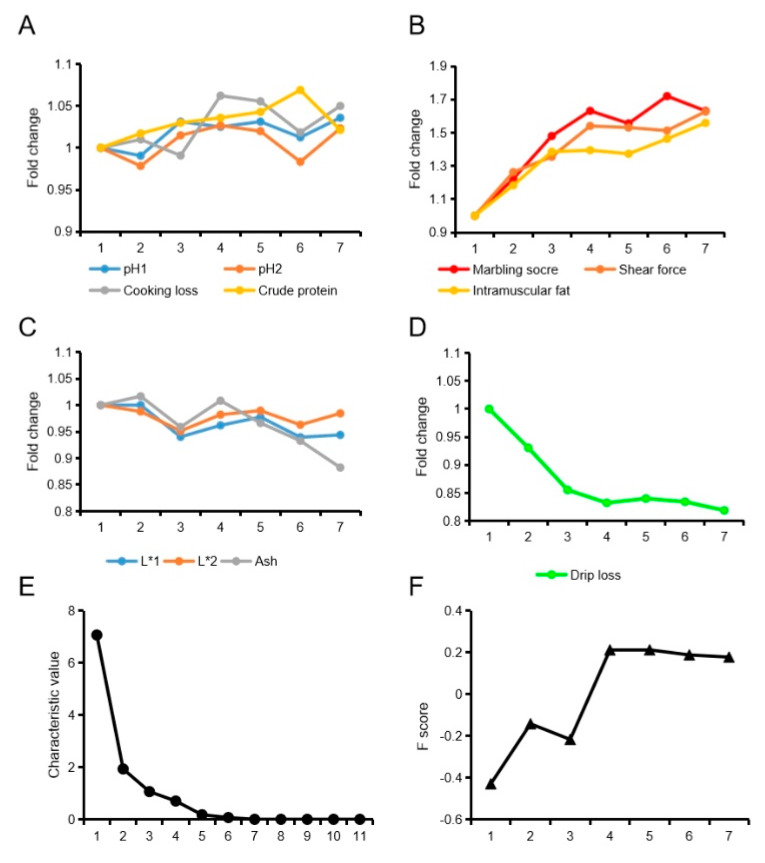
Change pattern of Liangshan pig meat quality traits. (**A**) Slowly increasing meat quality traits. (**B**) Rapidly increasing meat quality traits. (**C**) Slowly falling meat quality traits. (**D**) Rapidly falling meat quality traits. (**E**) Crushed stone graph for factor analysis of meat quality traits. (**F**) Comprehensive score for factor analysis of meat quality traits.

With the increase in slaughter weight, meat samples’ pH_1_, pH_2_, cooking loss, and crude protein increased slowly and fluctuated, while marbling score, shear force and intramuscular fat content rapidly and continuously increased (Figure 1A,B). As the slaughter weight increased, L*_1_, L*_2_ and ash decreased slowly and fluctuated, while drip loss rapidly and continuously decreased (Figure 1C,D). The overall analysis score for of Liangshan pig quality factors first increased and then remained at high levels with further increases in bodyweight (Figure 1E,F).

### 3.2. Free Amino Acid Contents

**Figure 2:**
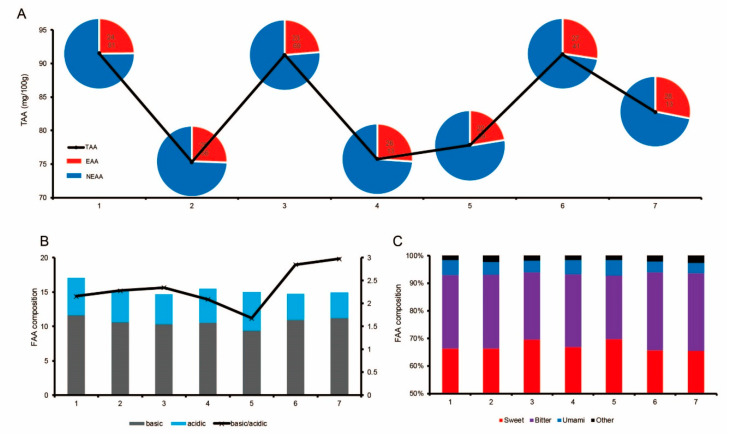
Analysis of amino acid composition in the longissimus dorsi muscle. (**A**) Changes in total amino acid (TAA) content and changes in essential amino acids (EAA) and non-essential amino acids (NEAA). (**B**) The content of basic and acidic amino acids. (**C**) The amino acid ratios of longissimus dorsi muscle with different flavors. Umami AA: Glu, Asp; Sweet AA: Gly, Ala, Ser, Thr, Pro, Gln, Lys; Bitter AA: Tyr, Arg, His, Val, Met, Ile, Leu, Trp, Phe.

It can be seen in Table 3 and Figure 2A that the total amino acid (TAA) content in the longissimus dorsi muscle of Liangshan pigs slightly fluctuated (˂30%) at different stages; the highest value was in the first stage and the lowest was in the second stage. Lys, Ile, Val, Trp, His, Arg, Glu, Tyr and Ala contents in Liangshan pig longissimus dorsi muscle were highest in the first stage, and Thr, Gln, Gly and Pro were the highest in the third stage (Table 3). Leu, Met, Ser and Asp contents were highest in the sixth stage, and Ile and Asn were the highest in the seventh stage.

The content of essential amino acids (EAA) in the longissimus dorsi muscle of Liangshan pigs in the seventh stage was the highest, reaching 28.13% (Figure 2A). Further analysis revealed that as the slaughter weight increased, the composition of basic amino acids and acidic amino acids in the longissimus dorsi muscle showed a downward trend and fluctuated, while the ratio of basic amino acids to acidic amino acids increased in fluctuation (Figure 2B). Sweet and umami amino acids were highest in the fifth stage, while bitter amino acids were highest in the sixth stage (Figure 2C).

### 3.3. Fatty Acid Levels

**Figure 3:**
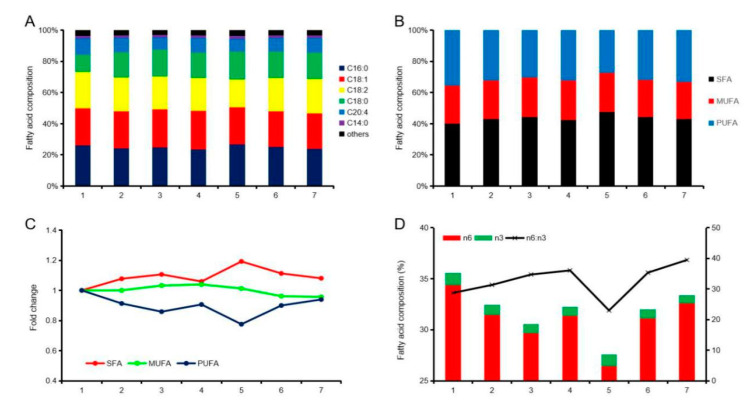
Analysis of fatty acid composition and characteristics in the longissimus dorsi muscle. (**A**) The content of the top 6 fatty acids. (**B**) A composition of saturated fatty acids (SFA), monounsaturated fatty acids (MUFA) and polyunsaturated fatty acids (PUFA) in longissimus dorsi muscle. (**C**) Changes in SFA, MUFA and PUFA contents. (**D**) The ratio of n6:n3 of longissimus dorsi muscle.

A total of 24 fatty acids were measured in the longissimus dorsi muscle of Liangshan pigs in the seven stages tested (Table 4). C16:0, C18:1, C18:2, C18:0, C20:4 and C14:0 are contained in more than 1%, and the cumulative proportion of these fatty acids exceeded 96% in the seven stages (Figure 3A). The C18:1 content was highest in the fourth stage, and the C16:0 content was highest in all stages other than stage six. The saturated fatty acids (SFA) was the lowest in the first stage and the highest in the fifth stage. Monounsaturated fatty acid (MUFA) content was highest in the fourth stage and lowest in the seventh stage. Polyunsaturated fatty acids (PUFA) content was highest in the first stage and lowest in the fifth stage (Figure 3B,C). Further analysis revealed that the overall n6 and n3 content showed a downward trend, while the n6:n3 values increased in volatility (Figure 3D).

### 3.4. Comprehensive Meat Quality Evaluation of Liangshan Pigs at Different Bodyweight Stages

Correlation analysis was performed on meat quality indicators that changed by >50% of their values at the first stage. Mar bling score was significantly positively correlated with shear force, intramuscular fat, and C18:0 content. Shear force was significantly positively correlated with intramuscular fat and C18:0 content. Intramuscular fat content was significantly positively correlated with Asp and C18:0 content, and significantly negatively correlated with C22:6 content (Table 5).

**Figure 4:**
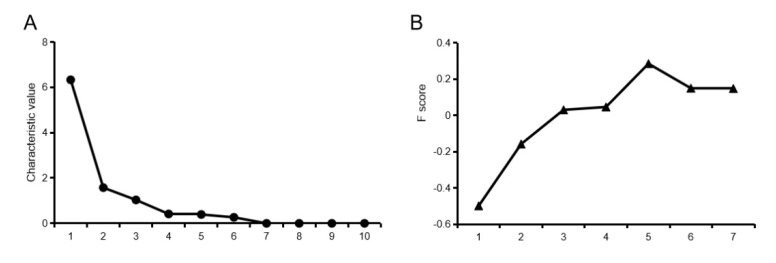
Results of the factor analysis. (**A**) A crushed stone graph. (**B**) A comprehensive score for factor analysis.

Through factor analysis, three characteristic values greater than 1 were obtained, and the cumulative contribution rate of the components reached 91.18% (Figure 4A). The component matrix results are shown in Table 6. The first principal component (PC) is mainly related to mar bling score, shear force and C18:0 content, the second principal component is mainly related to C22:6 content, and the third principal component is mainly related to C20:2 content (Table 6). As can be seen from Figure 4B, the comprehensive score shows a trend of first increasing, then decreasing (Figure 4B).

## 4. Discussion

The Liangshan pig is a typical small-sized mountain-type pig breed, which is mainly distri buted in the Yi Autonomous Prefecture of Liangshan. Like most of the world’s local pig breeds, Liangshan pigs are endangered [10,11]. The natural environment of the Liangshan Yi area and the dietary culture of the local people have determined the characteristics of Liangshan pigs. However, little is known about the basic biological characteristics of the Liangshan pig. Many studies have shown that age and weight are the most important factors affecting meat quality [12,13]. Here, meat quality traits, and amino acid and fatty acid composition of the longissimus dorsi muscle of Liangshan pigs were measured at seven stages (between 50 and 90 kg bodyweight) and were analyzed for their characteristics and changes.

Studies on the development of animal tissues and organs have shown that fat deposition occurs later than muscle deposition, and fat deposition is rapid after the turning point in animal growth [14]. Our previous results showed that the Liangshan pig growth turning point was 193.4 days at 62.5 kg [6]. In the present study, mar bling score and IMF content increased rapidly with weight gain. As Liangshan pigs’ weight increased, the shear force of the longissimus dorsi muscle rapidly increased, which may be due to the gradual growth of the muscle fi ber diameter and an increase in muscle connective tissue content [15]. Drip loss decreased as slaughter weight increased. The effect of weight on drip loss is consistent with the findings of other reports [13,16].

Amino acids are basic units that make up proteins required by animals [17]. EAAs must be o btained directly from food, which is extremely important for maintaining the body’s nitrogen balance and health [18]. The total amount of amino acids in the longissimus dorsi muscle of Liangshan pigs at different weights remained relatively stable, but EAA content showed an upward trend. Amino acid composition is also related to the taste of meat. Amino acids are normally divided into sweet amino acids, bitter amino acids, and umami amino acids [19,20]. The sweet and bitter amino acid contents of Liangshan pigs were relatively sta ble at different bodyweights, but umami amino acid content gradually decreased. Approximately 30% of umami amino acids were lost by the seventh stage compared to the first stage.

Amino acids are also divided into neutral amino acids, basic amino acids, and acidic amino acids [21]. The acidity and basicity of amino acids are usually determined according to the num ber of car boxyl groups and amino groups. Amino acids with more car boxyl groups than amino groups per molecule are termed ‘acidic’ (Asp and Glu) [22], otherwise they are termed ‘basic’ (Arg, Lys and His) [23]. Interestingly, as the weight of Liangshan pigs increased in this study, basic and acidic amino acid contents decreased, while the ratio of basic to acidic amino acids increased. This may be a reason for the increase in Liangshan pig meat sample pH as weight increased.

As bodyweight increased, the total fatty acid content of Liangshan pig meat samples showed an upward trend, which was consistent with intramuscular fat content and mar bling score. Dietary fatty acids are closely related to cardiovascular health, and higher SFA content in meat products has been shown to affect cholesterol metabolism [24]. PUFAs possess many physiological functions [25], such as maintaining biofilm structures, treating cardiovascular diseases [26], anti-inflammation [27], and the promotion of brain development [28]. It is worth noting that as the weight of Liangshan pigs increased, SFA content showed an upward trend, while the changes of PUFA were symmetrical with SFA. Further analysis revealed that n6:n3 values in Liangshan pig meat samples showed a rising trend of volatility.

Among the 54 indicators measured in this study, 10 indicators changed by more than 50%. Among these indicators, three were of meat quality traits, four were amino acids, and three were fatty acids. Correlation analysis showed that as intramuscular fat was deposited, C18:0 and Asp content increased rapidly, while C22:6 content decreased rapidly. Although saturated fatty acids are associated with a higher risk of cardiovascular disease, C18:0 does not lead to an increase in blood cholesterol [24]. Asp is an umami amino acid, and an increase in Asp can improve the taste of pork [29]. Further factor analysis shows that, in the fourth to seventh stages, the comprehensive score was higher, which is consistent with the factor analysis results based on meat quality traits. A similar pattern was also found in other pig breeds [16].

## 5. Conclusions

The current results show that differences in meat quality, amino acid composition, and fatty acid composition are present in Liangshan pigs at different slaughter weights. As bodyweight increased, mar bling score, intramuscular fat, shear force, Met, Asp, Asn, C18:0, and C20:2 content increased, and drip loss, Trp and C22:6 content decreased. The comprehensive factor score first increased and then decrease with weight gain within 74.9–91.5 kg of bodyweight. When slaughtering between 74.9 and 80.4 kg, the meat quality of Liangshan pigs is the best. Slaughtering between 74.5 and 80.4 kg provides the best meat quality in Liangshan pigs. In addition, when slaughtered at 80.4 kg, pork had the highest sweet amino acid content and the lowest n6:n3 ratio. Therefore, considering the meat quality, amino acid composition and fatty acid composition, the suitable slaughter weight of Liangshan pigs is 74.9–80.4 kg. This study provides effective data for the genetic improvement and specialty food processing of Liangshan pigs and provides new insights and references for research into local high incidences of disease.

## Figures and Tables

**Table 1:** Information of Liangshan pigs being slaughtered.

Group.	1	2	3	4	5	6	7
Number	20	20	20	20	20	20	20
Body weight, kg	53.2	59.5	67.4	74.9	80.4	86.7	91.5

**Table 2:** Meat quality of Liangshan pig at different stages.

Meat Quality	Group							S.E.	Significance
	1	2	3	4	5	6	7		
pH_1_	6.40 ^b^	6.34 ^b^	6.60 ^ab^	6.56 ^ab^	6.60 ^ab^	6.48 ^b^	6.63 ^a^	0.04	*
pH_2_	6.02 ^b^	5.89 ^b^	6.11 ^ab^	6.18 ^a^	6.14 ^ab^	5.92 ^b^	6.16 ^ab^	0.04	*
L*_1_	41.49 ^a^	41.50 ^a^	39.01 ^b^	39.91 ^b^	40.56 ^ab^	38.98 ^b^	39.16 ^b^	0.42	*
L*_2_	44.58 ^a^	44.05 ^a^	42.41 ^a^	43.78 ^ab^	44.12 ^a^	42.93 ^b^	43.89 ^ab^	0.28	*
Marbling score	2.25 ^b^	2.75 ^b^	3.33 ^ab^	3.67 ^a^	3.50 ^a^	3.87 ^a^	3.67 ^a^	0.22	*
Shear force, kg	3.24 ^b^	4.09 ^b^	4.39 ^ab^	4.99 ^ab^	4.96 ^ab^	4.90 ^ab^	5.27 ^a^	0.27	*
Drip loss, %	5.18 ^a^	4.82 ^ab^	4.43 ^b^	4.31 ^b^	4.35 ^b^	4.32 ^b^	4.24 ^b^	0.13	*
Cooking loss, %	34.05 ^b^	34.39 ^b^	33.74 ^b^	36.17 ^a^	35.94 ^ab^	34.68 ^b^	35.75 ^ab^	0.37	*
Crude protein, %	18.69 ^c^	19.01 ^bc^	19.25 ^b^	19.36 ^b^	19.49 ^ab^	19.98 ^a^	19.09 ^bc^	0.15	*
Intramuscular fat, %	3.22 ^b^	3.81 ^b^	4.46 ^ab^	4.49 ^ab^	4.42 ^ab^	4.71 ^a^	5.02 ^a^	0.76	*
Ash, %	1.19 ^b^	1.21 ^b^	1.16 ^b^	1.20 ^b^	1.15 ^b^	1.11 ^b^	1.05 ^a^	0.27	*

pH_1_ and L*_1_ measured at 45 min postmortem; pH2 and L*_2_ measured at 24 h postmortem. S.E. standard error, ^a^, ^b^, ^c^ and * mean significant difference.

**Table 3:** The amino acid content of longissimus dorsi muscle of Liangshan pig (mg/100g).

Amino Acid	Phase							S.E.	Significance
	1	2	3	4	5	6	7		
**EAA**									
Lys	4.11 ^a^	3.04 ^b^	3.95 ^a^	3.22 ^b^	2.90 ^b^	3.71 ^ab^	3.96 ^a^	0.19	*
Ile	2.39 ^b^	2.13 ^bc^	2.27 ^b^	2.07 ^b c^	1.73 ^c^	2.92 ^a^	2.39 ^b^	0.14	*
Leu	4.09 ^b^	3.54 ^bc^	3.82 ^bc^	3.70 ^bc^	3.13 ^c^	5.25 ^a^	4.27 ^b^	0.25	*
Val	4.05 ^a^	3.30 ^bc^	3.56 ^b^	3.47 ^b^	3.01 ^c^	3.33 ^bc^	3.67 ^ab^	0.12	*
Thr	3.09 ^ab^	2.64 ^b^	3.22 ^a^	2.68 ^b^	2.44 ^b^	3.57 ^a^	3.05 ^ab^	0.15	*
Phe	2.79 ^b^	2.75 ^b^	2.81 ^ab^	2.43 ^b^	2.46 ^b^	3.19 ^a^	3.10 ^ab^	0.11	*
Met	1.62 ^b^	1.49 ^b^	1.51 ^b^	1.76 ^b^	1.55 ^b^	2.80 ^a^	2.61 ^a^	0.21	*
Trp	0.57 ^a^	0.35 ^b^	0.48 ^a b^	0.46 ^ab^	0.21 ^b^	0.28 ^b^	0.23 ^b^	0.05	*
**NEAA**									
His	3.17 ^a^	2.62 ^b^	2.79 ^b^	2.52 ^b^	2.55 ^b^	3.09 ^ab^	2.60 ^b^	0.10	*
Gln	19.76 ^bc^	19.07 ^bc^	25.13 ^a^	17.88 ^c^	21.23 ^b^	22.31 ^ab^	18.90 ^bc^	0.94	*
Arg	3.38 ^a^	2.31 ^bc^	2.66 ^b^	2.22 ^bc^	1.87 ^c^	3.19 ^ab^	2.72 ^ab^	0.20	*
Glu	4.60 ^a^	3.22 ^bc^	3.52 ^b^	3.48 ^b^	4.03 ^b^	3.01 ^bc^	2.63 ^c^	0.25	*
Ser	3.71 ^ab^	2.84 ^b^	3.04 ^b^	2.91 ^b^	2.84 ^b^	4.52 ^a^	4.14 ^a^	0.26	*
Asp	0.34 ^b^	0.27 ^b^	0.48 ^a^	0.32 ^b^	0.33 ^b^	0.50 ^a^	0.49 ^a^	0.04	*
Gly	7.70 ^ab^	6.29 ^b^	7.88 ^a^	6.01 ^b^	6.77 ^b^	6.25 ^b^	6.02 ^b^	0.30	*
Tyr	2.36 ^a^	1.65 ^b^	2.34 ^a^	1.39 ^b^	1.50 ^b^	1.94 ^ab^	1.72 ^b^	0.15	*
Ala	21.39 ^a^	15.17 ^c^	19.15 ^ab^	17.16 ^bc^	17.08 ^bc^	18.57 ^b^	17.04 ^bc^	0.75	*
Asn	1.52 ^b^	1.68 ^b^	1.59 ^b^	1.25 ^b^	1.26 ^b^	1.94 ^ab^	2.17 ^a^	0.13	*
Pro	0.89 ^b^	0.92 ^b^	1.06 ^a^	0.81 ^b^	0.94 ^ab^	1.01 ^ab^	1.05 ^a^	0.03	*
**TAA**	91.53 ^a^	75.28 ^b^	91.26 ^a^	75.74 ^b^	77.83 ^b^	91.38 ^a^	82.76 ^ab^	2.87	*

^a^, ^b^, ^c^ and * mean significant difference.

**Table 4:** Fatty acid content of the longissimus dorsi muscle of the Liangshan pig (mg/100g).

FA	Phase							S.E.	Significance
	1	2	3	4	5	6	7		
C8:0	0.25 ^b^	0.25 ^b^	0.21 ^b^	0.31 ^a^	0.30 ^ab^	0.30 ^ab^	0.34 ^a^	0.02	
C10:0	2.39 ^b^	2.56 ^b^	2.33 ^b^	3.50 ^a^	3.19 ^a^	3.21 ^a^	3.24 ^a^	0.18	*
C12:0	1.93 ^b^	2.23 ^b^	1.74 ^b^	2.67 ^a^	2.89 ^a^	2.63 ^a^	2.47 ^a^	0.16	*
C14:0	24.95 ^b^	29.87 ^a^	23.21 ^b^	33.81 ^a^	33.98 ^a^	34.43 ^a^	32.08 ^a^	1.73	*
C15:0	1.23 ^c^	1.37 ^bc^	1.24 ^c^	1.47 ^bc^	1.85 ^a^	1.43 ^bc^	1.54 ^b^	0.08	*
C15:1	0.69 ^b^	0.88 ^ab^	0.69 ^b^	0.68 ^b^	0.69 ^b^	1.02 ^a^	1.10 ^a^	0.07	*
C16:0	391.56 ^b^	453.18 ^b^	397.11 ^b^	491.12 ^ab^	517.62 ^ab^	534.75 ^a^	490.13 ^ab^	21.29	*
C17:0	4.18 ^b^	5.02 ^b^	4.31 ^b^	5.47 ^ab^	6.07 ^a^	5.36 ^ab^	5.84 ^ab^	0.27	*
C17:1	3.03 ^b^	4.26 ^ab^	3.69 ^b^	4.62 ^ab^	5.03 ^a^	4.78 ^ab^	3.92 ^b^	0.26	*
C18:0	165.32 ^b^	301.69 ^a^	275.60 ^a^	337.06 ^a^	350.95 ^a^	356.85 ^a^	344.99 ^a^	25.73	*
C18:1	353.17 ^b^	440.08 ^ab^	393.2 ^b^	513.13 ^a^	463.96 ^ab^	482.58 ^a^	464.37 ^ab^	19.75	*
C18:2	349.93 ^b^	403.99 ^ab^	340.86 ^b^	443.20 ^a^	341.58 ^b^	458.11 ^a^	455.98 ^a^	20.59	*
C18:3	6.71 ^b^	7.96 ^a^	6.45 ^b^	8.76 ^a^	9.12 ^a^	9.16 ^a^	8.04 ^ab^	0.42	*
C20:0	3.34 ^b^	4.12 ^ab^	3.48 ^b^	4.74 ^a^	4.98 ^a^	4.91 ^a^	4.91 ^a^	0.27	*
C20:1	8.40 ^b^	10.55 ^ab^	8.40 ^b^	11.77 ^a^	10.96 ^ab^	12.16 ^a^	11.72 ^a^	0.59	*
C20:2	9.33 ^b^	12.16 ^b^	9.54 ^b^	13.15 ^ab^	13.10 ^ab^	13.12 ^ab^	15.47 ^a^	0.82	*
C20:3	1.45 ^ab^	1.42 ^ab^	0.99 ^c^	1.39 ^ab^	1.31 ^b^	1.41 ^ab^	1.61 ^a^	0.07	*
C20:4	153.91 ^b^	169.97 ^ab^	126.87 ^b^	197.36 ^a^	158.14 ^b^	190.68 ^ab^	198.31 ^a^	10.04	*
C20:5	3.11 ^bc^	3.31 ^b^	2.60 ^c^	3.30 ^b^	4.07 ^a^	3.46 ^b^	3.16 ^bc^	0.17	*
C22:0	0.93 ^b^	0.90 ^b^	0.79 ^b^	1.01 ^b^	1.36 ^a^	1.03 ^b^	1.03 ^b^	0.07	*
C22:1	0.54 ^b^	0.60 ^b^	0.54 ^b^	0.57 ^b^	0.75 ^a^	0.56 ^b^	0.57 ^b^	0.03	*
C22:6	6.61 ^ab^	6.02 ^ab^	3.71 ^b^	4.68 ^b^	7.82 ^a^	4.70 ^b^	4.16 ^b^	0.56	*
C23:0	0.14 ^b^	0.12 ^b^	0.10 ^b^	0.15 ^b^	0.26 ^a^	0.13 ^b^	0.14 ^b^	0.02	*
C24:0	0.51 ^b^	0.48 ^b^	0.41 ^b^	0.60 ^b^	0.93 ^a^	0.54 ^b^	0.62 ^b^	0.06	*
TFA	1493.61 ^b^	1862.99 ^ab^	1608.07 ^b^	2084.52 ^a^	1940.91 ^ab^	2127.31 ^a^	2055.74 ^a^	92.93	*

^a^, ^b^, ^c^ and * mean significant difference.

**Table 5:** Correlation analysis of the indexes of longissimus dorsi in Liangshan pig.

	MS	SF	IMF	Trp	Met	Asp	Asn	C18:0	C20:2	C22:6
MS	1									
SF	0.95 *	1								
IMF	0.95 *	0.95 *	1							
Trp	−0.58	−0.65	−0.64	1						
Met	0.66	0.67	0.7	−0.62	1					
Asp	0.71	0.64	0.83 *	−0.55	0.68	1				
Asn	0.23	0.35	0.43	−0.53	0.71	0.52	1			
C18:0	0.79 *	0.83 *	0.80 *	−0.64	0.29	0.46	0.22	1		
C20:2	0.22	0.48	0.41	−0.6	0.54	0.3	0.67	0.23	1	
C22:6	−0.74	−0.67	−0.8 *	0.19	−0.61	−0.75	−0.49	−0.53	−0.1	1

* mean significant difference.

**Table 6:** Rotated component matrix.

PC ^1^	MS	SF	IMF	Trp	Met	Asp	Asn	C18:0	C20:2	C22:6
1	0.811	0.833	0.749	−0.636	0.244	0.382	−0.051	0.919	0.193	−0.333
2	0.546	0.415	0.602	−0.028	0.637	0.751	0.486	0.174	−0.004	−0.911
3	0.073	0.277	0.25	−0.676	0.609	0.286	0.8	0.082	0.901	−0.004

PC: principal component. ^1^: Only the principal components with feature values greater than 1 are displayed.

## Supplementary Materials

The following are available online at https://www.mdpi.com/2076-2615/10/5/822/s1, Table S1: Ingredients of the basal experiment diets.
