# Supplementary material for: Meat Quality, Amino Acid, and Fatty Acid Composition of Liangshan Pigs at Different Weights
Source: Animals (Basel). 2020 May 9;10(5):822. doi: 10.3390/ani10050822 (PMC7278381; doi:10.3390/ani10050822)
Supplement: Supplementary file 1 [file animals-10-00822-s001.pdf]

Table S1. Ingredients of the basal experiment diets.

Item	g/kg
Corn	772
Soybean	200
CaHPO4	8
$CaCO_3$	7
NaCl	3
Lysine	——
Premix	10
